# Spatial–temporal distribution characteristics and traceability analysis of organic matter in Shahe Reservoir (Beijing, China)

**DOI:** 10.1038/s41598-023-49060-x

**Published:** 2023-12-06

**Authors:** Sun Wen, Zhang Yang, Peng Biao, Wang Jing, Dian Liu

**Affiliations:** 1 Shaanxi Provincial Land Engineering Construction Group, Key Laboratory of Degraded and Unused Land Consolidation Engineering, Ministry of Natural Resources, Xi’an, 710075 China; 2 School of Energy and Power Engineering, Xi’an Jiaotong University, Xi’an, 710049 China; 3 Shaanxi Key Laboratory of Land Consolidation, Chang’an University, Xi’an, 710054 China; 4 China Communications Construction Second Harbor Consultants Co.,Ltd, Wuhan, 430060 China

**Keywords:** Environmental sciences, Environmental chemistry, Environmental impact

## Abstract

Shahe Reservoir is a key node in the upstream of the North Canal, and the water quality has gradually improved after the implementation of low water operation in 2018. The organic matter(OM) in the sediment decreased from 16.66 to 14.22%.In this study, the FI index and parallel factor method were used to investigate the traceability of OM and dissolved organic matter (DOM) in the Shahe Reservoir before and during low water level operation(LWLO), and the results showed that the terrestrial source fraction of OM in sediments was mainly related to organic-rich terrestrial plant residues carried by tributaries and overflows/outfalls during the rainy season, and the FI index indicated that the organic matter (OM) in the Shahe Reservoir before and during LWLO in each DOM in the area is derived from authigenic sources of autotrophic microorganisms, algae, etc. The parallel factor method shows that more than most of the pollutants in the DOM are input from endogenous sources and a small proportion of pollutants are input from exogenous sources. Nutrients in both sediment and interstitial water increased during the LWLO, with TN and TP levels increasing by 262.38 and 204.45 mg·kg^−1^ in sediment, NH_4_^+^–N, PO_4_^3−^−P, TN and TP in interstitial water increasing by 0.98, 1.36, 2.07 and 4.33 mg·L^−1^, respectively. Pearson correlation analysis and principal component analysis showed that OM was significantly correlated with nutrients: OM and TN (*p* < 0.01) and OM and TP (*p* < 0.05) in the pre-LWLO; OM and TN and TP (*p* < 0.01) in the LWLO.The results suggested that organic matter pollution control should be mainly carried out from the perspective of endogenous input, focusing on controlling the release of nutrients in sediments.

The North Canal is one of the five major water systems in Beijing, and it is the water system with the largest watershed area and the most tributaries in Beijing, flowing through Changping and Tongzhou districts of Beijing, and undertaking 90% of the drainage tasks in the central city of Beijing^1^. Therefore, the improvement of water environment quality and water ecology function of Shahe Reservoir is a key link to achieve the improvement of water quality in the upper reaches of the North Canal and the water system of Changping District^2^. Since June 2018, the Shahe reservoir has implemented low water level operation(LWLO), and the water level in the reservoir has been reduced from 35.7 to 34.1 m^3^, and with the implementation of the upstream source control and interception project, the water quality of the Shahe reservoir has been steadily improved.Dissolved organic matter is a complex organic compound, mainly composed of protein-like substances (like tryptophan, like tyrosine, etc.) and humic substances (like fulvic acid, etc.), which has an impact on the water environment. The role of nutrient transformation and carbon cycle^4,5^. DOM in natural water bodies mainly comes from terrestrial inputs such as humus-like substances in the soil, the degradation of dead animals and plants, and endogenous contributions such as the release of phytoplankton and the degradation of plankton residues^6^. Changes in river DOM are related to soil types around the basin, human activities, and urban sewage discharge^7^. In addition, the degradation of microorganisms in the water body will also cause changes in the DOM of rivers^8^.However, the content of organic matter (OM)and dissolved organic matter (DOM) in the Shahe reservoir is still high and its source is not clear . Therefore, a full understanding of the changing characteristics and source analysis of OM and DOM in the water in the reservoir area of Shahe Reservoir after LWLO is an important guidance for the subsequent eutrophication control and water ecology restoration of Shahe Reservoir.

Three-dimensional excitation emission matrix (3D-EEM) is a convenient, efficient and non-destructive method for DOM analysis, which can be monitored online in real time^9^ and has great potential in DOM traceability analysis. 3D fluorescence spectroscopy contains a large amount of fingerprint information from DOM, and its interpretation and screening of valid information is a difficult task in spectral interpretation and traceability analysis. Previous studies the seasonal water level fluctuations in reservoirs during the annual abundant, flat and dry seasons, and the associated water quality, microbial abundance changes and community succession^10–13^. However, the effects of hydrological processes on reservoir water quality and ecology, such as LWLO under artificial intervention conditions, are still poorly reported^14^.This paper addresses the need of DOM traceability for pollution control in the Shahe reservoir in the upper reaches of the North Canal, and adopts a three-dimensional fluorescence-based DOM spectral interpretation and analysis method to investigate the succession characteristics of DOM during the process of LWLO, clarify its important components, and analyze its sources and influencing factors. Through principal component analysis and Pearson correlation analysis, the correlation characteristics between nutrients and DOM were studied, providing a reference for organic matter control in typical river-type reservoirs.

## Materials and methods

### Brief mapping of the studied area

**Figure 1:**
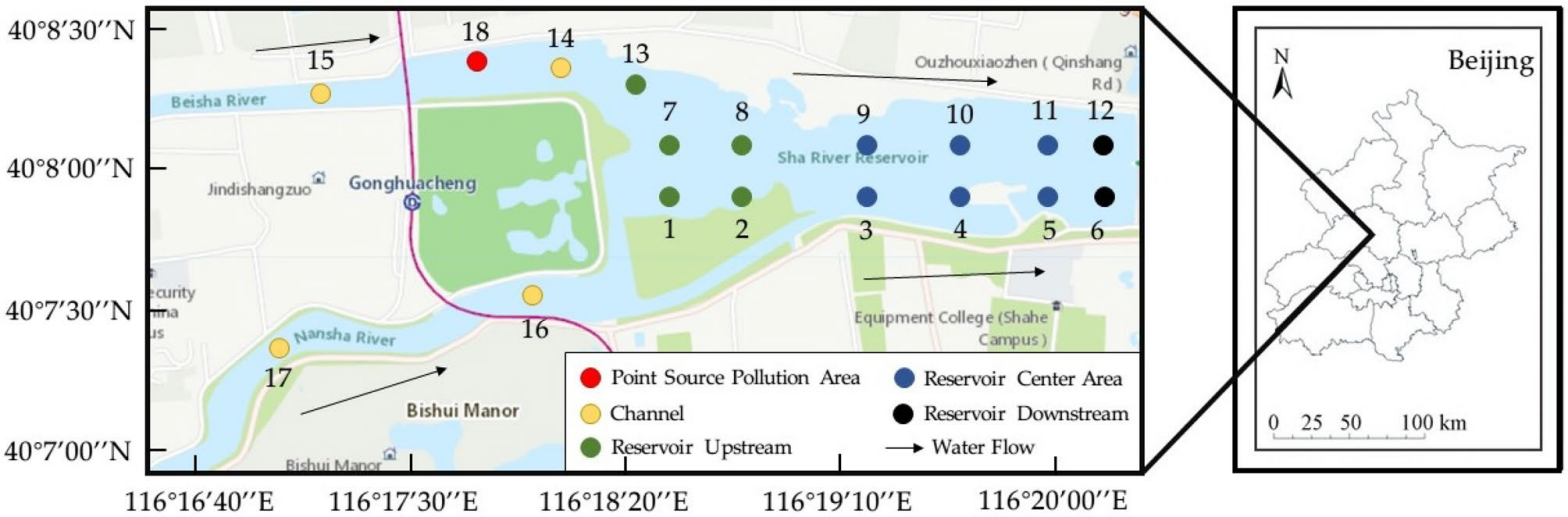
Sampling sites in the Shahe Reservoir (The figure wascreated by S.W. and modifed using ArcGIS sofware 10.2; *Source*: WGS 1984).

The North Canal water system originates at the southern foot of Yanshan Mountain in Changping District, Beijing, and flows through Beijing, Langfang, Hebei and Tianjin successively^15^.The Shahe Reservoir is an important node located in the source area of the Northern Canal. The watershed area of the Shahe Reservoir is about 1125 km^2^, of which the mountainous area accounts for about 75%. The Shahe Reservoir is a typical channel-type reservoir in the upper reaches of the North Canal. It is mainly recharged by the return water from urban wastewater treatment plants. The water in the Shahe reservoir area has a long residence time and is a stagnant body of water^16^. Eutrophication of reservoir water is a prominent problem. The Shahe reservoir started LWLO mode in June 2018 and the water depth in the reservoir area decreased nearly 2 m and the velocity of water flow increased^17,18^. The locations and zones of sediment and interstitial water sampling points are shown in Fig. 1 and Table 1.

**Table 1:** Sampling point coordinates and division in Shahe Reservoir.

Reservoir zoning	Sampling site	Longitude	Latitude
Reservoir upstream	S1	116°18′32.59″E	40°07′54.42″N
	S2	116°18′55.19″E	40°07′54.42″N
	S7	116°18′32.59″E	40°08′01.21″N
	S8	116°18′55.19″E	40°08′01.21″N
	S13	116°18′32.59″E	40°08′14.48″N
Channel	S14	116°20′12.36″E	40°08′16.84″N
	S15	116°17′37.24″E	40°08′11.99″N
	S16	116°18′06.18″E	40°07′35.68″N
	S17	116°17′14.51″E	40°07′29.13″N
Reservoir center area	S3	116°19′14.19″E	40°07′54.42″N
	S4	116°19′39.45″E	40°07′54.42″N
	S5	116°19′54.59″E	40°07′54.42″N
	S9	116°19′14.19″E	40°08′01.21″N
	S10	116°19′39.45″E	40°08′01.21″N
	S11	116°19′54.59″E	40°08′01.21″N
Reservoir downstream	S6	116°20′12.36″E	40°07′54.42″N
	S12	116°20′12.36″E	40°08′01.21″N
Point source pollution area	S18	116°17′55.90″E	40°08′18.13″N

### Sample collection and processing

#### Sediment sample collection

The 0–20 cm surface sediments were collected using a Peterson mud picker in November 2017 (ULWLO) and September 2018 (LWLO), respectively. The collected surface sediments were protected from light and preserved at low temperature and brought back to the laboratory for freeze-drying (FD-1A-50 freeze-dryer, Beijing Boomedecon Experimental Instruments Co., Ltd.), then pressed and dispersed with a glass rod to remove impurities such as gravels, shells and plant and animal remains, and ground with a mortar and sieved through 100 mesh sieve. At the same time, the mud samples were obtained from the mud hopper, mixed and loaded into 50 mL centrifuge tubes, centrifuged at 4000 r/min for 20 min to obtain the interstitial water, and stored under refrigeration at  − 4 °C.

#### Sediment samples analysis

Fresh samples of sediment were taken to determine water content and organic matter (expressed as Loss on Ignition, LOI) by drying method^19^. Total Nitrogen (TN) content and C/N values in the sediments were determined using an elemental analyzer^20^(Vario MAX cube type, Elementar). Total Phosphorus (TP) in sediments was extracted using the SMT (Standards, Measurements and Testing) method developed in the framework of the European Committee for Standard Testing^21^, where sediment samples were cauterized at 450 °C and shaken with 3.5 mol/L HCl for 16 h at room temperature. The TP content of the extracts was then determined by molybdenum antimony anti-spectrophotometric method^22^.

#### Interstitial water samples analysis

Nutrient indicators for interstitial water determination included ammonia nitrogen (NH_4_^+^-N), total nitrogen (TN), orthophosphate (PO_4_^3−^–P), total phosphorus (TP) and dissolved organic matter (DOM). Nitrogen and phosphorus indicators were analyzed with reference to the Analytical Methods for Water and Wastewater Monitoring.The interstitial water was diluted a reasonable number of times, and after potassium sulfate digestion (Alfa Aesar, UK), the UV spectrophotometer colorimetric method was used to detect TN, the molybdenum antimony anti-spectrophotometric method was usedto detect TP.The nano reagent spectrophotometric method was used to detect NH_4_^+^-N.The molybdenum antimony anti-spectrophotometric method was used todetect PO_4_^3-^–P.All the instruments used for the determination were UV–visible spectrophotometer (TU-1901, Beijing Pu-analysis General Instrument Co.)

#### 3D-EEM analysis

 The interpretation methods of 3D fluorescence spectra mainly include:

① *Peak Method*: The position and distribution relationship of typical peaks.
② *Fluorescence spectral characteristic parameters*: Such as Fluorescence Index (FI), Biogenic Index (BIX) and Humification Index (HIX), and other common indices^23^.
③ *Spectra decomposition*: Which uses mathematical and statistical methods to decompose the spectra into several types of components, is a widely used method, mainly Parallel Factor Analysis (PARAFAC) and its development of Parallel Factor Framework Clustering Analysis (PPFFCA), which is a statistical analysis method developed specifically for complex environmental samples such as wastewater. In addition, Principal Components Analysis (PCA) and chromatographic coupling are also methods for the interpretation of 3D fluorescence spectra.

The interstitial water samples were pre-filtered through a 0.45 μm bucket membrane and diluted with Milli-Q ultrapure water until the absorbance A was proposed to be < 0.05, and then the pre-treated water samples were spectroscopically scanned using a 3D fluorescence spectrometer (F-7000, Hitch Inc., Japan), with the light source of a 150W xenon lamp, the photomultiplier voltage of PMT = 700 V, and the excitation wavelength (λ_EX_) was 200–400 nm, and the emission wavelength (λ_Em_) was 220–550 nm,λ_EX_ and λ_Em_ slit width was set to 5 nm and the scanning speed was 12,000 nm·min^−1^. Milli-Q ultrapure water was used as a blank to remove the effect of Raman scattering from the water, and the toolkit removescater^24^was used on Matlab 2016b software to remove Raman scattering and Rayleigh Scattering.

#### Fluorescence index analysis

The fluorescence index FI can characterize the source of dissolved organic matter in water and is defined as the ratio of emission wavelengths at 470 and 500 nm when the excitation wavelength is 370 nm.

1 $$ {\text{FI}} = {\uplambda }_{470} /{\uplambda }_{520} $$

#### PARAFAC analysis

Parallel Factor Analysis (PARAFAC) was performed on the sample data using the DOMFluorvl I7 toolkit in Matlab 2016b software^25^.The PARAFAC method is based on the theory of trilinear analysis and employs the alternating least squares method as an The PARAFAC method is based on the theory of trilinear analysis and adopts the alternating least squares method, which is widely used in the analysis of three-dimensional and multidimensional data^26^, and the analysis steps include: deducting the blanks, removing the Raman and Rayleigh scattering, removing the outliers of fluorescence data, determining and verifying the number of fluorescence components, verifying the residual analysis, half-folding, and plotting contour lines and their corresponding parallel factor maps.

## Results and discussion

### Effect of LWLO on nutrients in Shahe Reservoir

**Figure 2:**
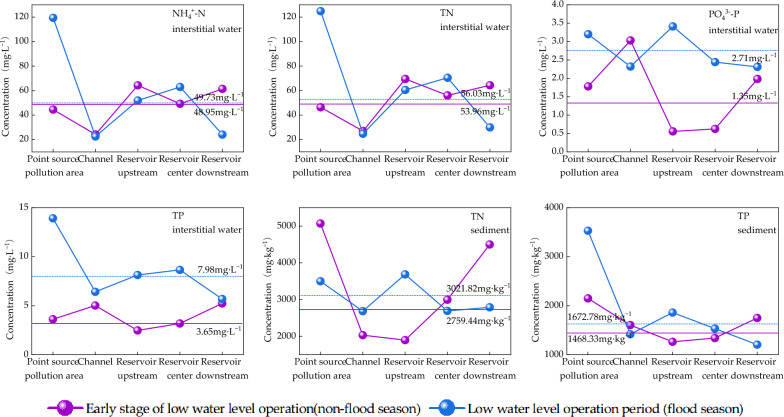
Distribution of pollutants in the LWLO period of Shahe Reservoir.

From July to August 2018, Shahe Reservoir entered the flood season and began to operate in low water mode, with an increase in reservoir flow velocity and a decrease in water depth of nearly 2 m from normal (~ 7 m). The effect of such a large and high flow rate of LWLO on the pollutants such as nitrogen and phosphorus in the Shahe Reservoir is shown in Fig. 2. NH_4_^+^–N and TN in the interstitial water of the Shahe Reservoir are similar, with mean values increasing by 0.78 and 2.34 mg·L^−1^respectively during LWLO compared to normal water level.The changes are mainly manifested in a large increase in the point source pollution zone (69.88, 78.43 mg·L^−1^), a small increase in the reservoir core zone (13.71, 13.92 mg·L^−1^), and a slight decrease in the river channel ( − 1.56,  − 1.85 mg·L^−1^). NH_4_^+^–N and TN remained basically unchanged in the river channel ( − 1.56,  − 1.85 mg·L^−1^), with a slight decrease in the upstream of the reservoir ( − 12.34,  − 8.97 mg·L^−1^) and a significant decrease in the downstream of the reservoir ( − 37.33,  − 34.39 mg·L^−1^).

PO_4_^3−^–P and TP in the interstitial water were about 1 times higher during LWLO than during normal water operation, with an increase of 1.36 and 4.33 mg·L^−1^, respectively; while the changes of PO_4_^3−^–P and TP in the interstitial water were slightly different. PO_4_^3−^–P mainly showed a small increase in the point source pollution area (1.42 mg·L^−1^), and the downstream of the reservoir (0.33 mg·L^−1^), while a large increase in the core area of the reservoir (1.82 mg·L^−1^) and the upstream of the reservoir (2.85 mg·L^−1^).The river channel ( − 0.71 mg·L^−1^) slightly decreased.TP mainly shows an increase in the whole reservoir area. Among them, the increase in point source pollution area (10.30 mg·L^−1^) is significant, and the increase of TP in the upstream (5.65 mg·L^−1^) and the core (5.47 mg·L^−1^) of the reservoir was obviously, and the increase of TP in the channel (1.40 mg·L^−1^) and downstream of the reservoir (0.46 mg·L^−1^)was slight.

TN and TP in the surface sediments of the Shahe Reservoir also increased during the LWLO, with specific increases of 262.38 and 204.45 mg·kg^−1^, respectively. TN mainly showed a significant increase in the upstream of the reservoir (1787.44 mg·kg^−1^), a small increase in the channel (651.72 mg·kg^−1^), and a slight decrease in the center of the reservoir ( − 305.10 mg·kg^−1^). The change of TP is reflected in a significant increase in the point source pollution area (1380.00 mg·kg^−1^), a slight increase in the upper reservoir (596.00 mg·kg^−1^) and the core area (193.33 mg·kg^−1^), and a slight decrease in the lower reservoir ( − 545.00 mg·kg^−1^) and the channel ( − 187.50 mg·kg^−1^).

The increase in the content of nitrogen and phosphorus pollutants in the interstitial water and sediment during the LWLO of the Shahe reservoir may be due to the fact that the LWLO mode (July to August 2019) is in the flood season (June to September) in Beijing, so there is a large input of exogenous pollution such as rainwater, pipeline overflow, and surface runoff. The changes in the horizontal space are mostly manifested in the increase of pollution load in the point source pollution area, which is caused by the overflow of rainwater and pipelines, and the significant increase of load in the reservoir core area, which is probably due to the slower water velocity in the reservoir core area for the deposition of pollutants. The downstream of the Shahe reservoir mostly shows a trend of pollution load reduction because the water flow velocity increases significantly when the Shahe gate is opened and released, and the strong hydrodynamic conditions lead to the release of pollutants such as nitrogen and phosphorus from the sediment, thus reducing the pollution load in the surface sediment and interstitial water.

### Spatial and temporal distribution of sediment organic matter and C/N values and traceability analysis

As shown in Table 2, the OM content and C/N values in the sediment of Shahe reservoir before the LWLO ranged from 2.99 to 31.03% and 8.18–18.73%, with mean values of 8.33 ± 6.18% and 11.28 ± 2.61%, respectively, and the highest content of 31.03% and 18.73% in S18 the point source pollution area and S13 the upstream of the reservoir. The OM content and C/N value of sediment in Shahe reservoir during LWLO ranged from 1.97 to 14.53% and 8.28–20.92%, with mean values of 7.11 ± 3.29% and 11.71 ± 3.46%, respectively, and the highest content in S2 and S13 the upstream of the reservoir, reaching 14.53% and 20.92%.

**Table 2:** Statistics of C/N ratio and Organic matter of the sediments.

	Sampling site								
Indicators	S1	S2	S3	S4	S5	S6	S7	S8	S9
OM(%,ULWLO)	10.11	6.79	12.37	2.99	5.15	9.45	5.72	3.18	9.92
OM(%,LWLO)	9.98	14.53	4.92	7.37	4.73	7.97	11.74	2.78	9.29
C/N(ULWLO)	9.80	10.16	10.18	14.84	11.09	8.43	11.71	14.35	8.90
C/N(LWLO)	8.56	8.72	14.90	8.83	9.75	8.60	8.70	17.72	8.28

	Sampling site								
Indicators	S10	S11	S12	S13	S14	S15	S16	S17	S18
OM(%,ULWLO)	7.72	9.91	10.78	4.19	7.72	4.13	4.69	4.09	31.03
OM(%,LWLO)	6.37	5.37	4.66	1.97	8.28	4.82	4.11	7.11	12.02
C/N(ULWLO)	9.98	8.49	8.18	18.73	11.36	11.80	13.58	11.65	9.89
C/N(LWLO)	9.43	12.26	15.21	20.92	10.76	12.98	13.11	10.83	11.18

**Figure 3:**
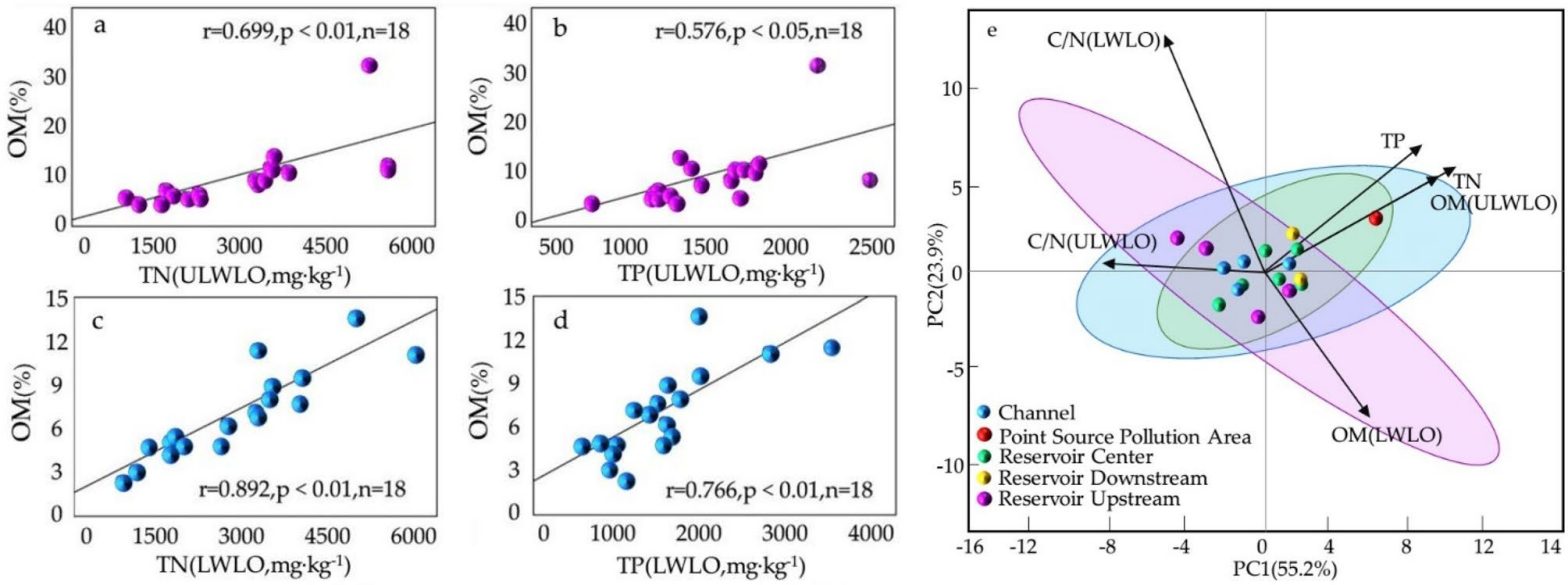
(**a**–**d**) Pearson correlation analysis of organic matter with TN and TP in sediment, **e** Principal component analysis of TN, TP, organic matter and each sampling site.

The results of Pearson correlation analysis of OM in sediment with TN and TP (Fig. 3a–d) showed that OM in sediment before the LWLO had a highly significant positive correlation with TN (r = 0.699, *p* < 0.01) and a significant positive correlation with TP (r = 0.567, *p* < 0.05), while OM in sediment during the LWLO had a highly significant positive correlation with TN (r = 0.892, *p* < 0.01) and TP (r = 0.766, *p* < 0.01). TN (r = 0.892, *p* < 0.01) and TP (r = 0.766, *p* < 0.01) in the sediment at the LWLO had a highly significant positive correlation. It indicates that most of the TN and TP in the sediment may originate from organic nitrogen and organic phosphorus fractions in the OM, or particulate nitrogen and phosphorus adsorbed to the OM fraction. The correlation between nitrogen and phosphorus and OM suggests that they have a common source.

The principal component analysis (PCA) of OM and nutrient salts in the sediments before and during the LWLO was performed at each sampling site (Fig. 3e), and it was found that the channel, the upstream of the reservoir and the core area of the reservoir showed significant variability, and the OM and nutrient salts in different areas showed some variability, which may be caused by disturbed hydrodynamic conditions, temperature, and other factors. Accelerated water velocities during low-water operation resulted in the accumulation of nutrients from the upper reaches of the North Canal to the Shahe Reservoir, while hydrodynamic perturbations resulted in the release of nutrients from the sediments into the overlying water. It was found^27–29^that the effect of temperature on phosphorus release from the substrate was mainly realized through Ca–P, Fe–P and Org-P. On the one hand, elevated temperature can enhance the mineralization of sediment, which can increase the conversion of Ca–P and Org-P release; on the other hand, elevated temperature leads to an increase in oxygen consumption and a decrease in DO, so that the environment from oxidation to reduction, which is conducive to the conversion of Fe^3+^ to Fe^2+^, and promotes the release of Phosphorus from Fe–P in the sediment. Like phosphorus, the release of nitrogen is also affected by temperature to a certain extent^30^. With the increasing temperature, the microbial activity of the mud is strong, organic nitrogen is mineralized and decomposed into ammonia nitrogen, resulting in the release of ammonia nitrogen from the sediment is increasing; on the other hand, the mineralization of organic matter is accelerated, the oxygen is rapidly consumed, the reducing environment in the substrate so that the nitrification is inhibited, so that the overlying water NO^3-^-N is constantly diffused to the substrate.

It has been shown^31,32^that the C/N values of the sediment can be a useful indicator of the source of OM. The total organic carbon to nitrogen (C/N) ratio of OM from different sources differs significantly, with terrestrial higher plants having C/N values ranging from 14 to 23% or even greater than 30%, while lake plankton have C/N values of 6% to 7%, thus high C/N values tend to indicate a larger proportion of terrestrial organic matter in lake sediments^33–35^. In contrast, the mean C/N value of Shahe Reservoir before LWLO was 11.28 ± 2.61%, of which 9.89% was in the point source pollution zone, 12.10 ± 0.87% in the river, 12.95 ± 3.30% in the upper reservoir, 10.58 ± 2.08% in the core zone, and 8.31 ± 0.12% in the lower reservoir; the mean C/N value of Shahe Reservoir during LWLO was 11.71 ± 3.46%, of which 11.18% in the point source pollution area, 11.92 ± 1.13% in the river, 12.92 ± 5.32% in the upper reservoir, 10.57 ± 2.30% in the reservoir core area, and 11.90 ± 3.30% in the lower reservoir; this indicates that the OM in the sediment of all areas of the Shahe reservoir is characterized by mixed sources. Among them, the terrestrial source fraction of OM in the sediment is mainly related to the organic-rich terrestrial plant residues carried by tributaries and overflow/outfall rainy seasons.

### Spatial and temporal distribution and traceability analysis of DOM in interstitial water

**Figure 4:**
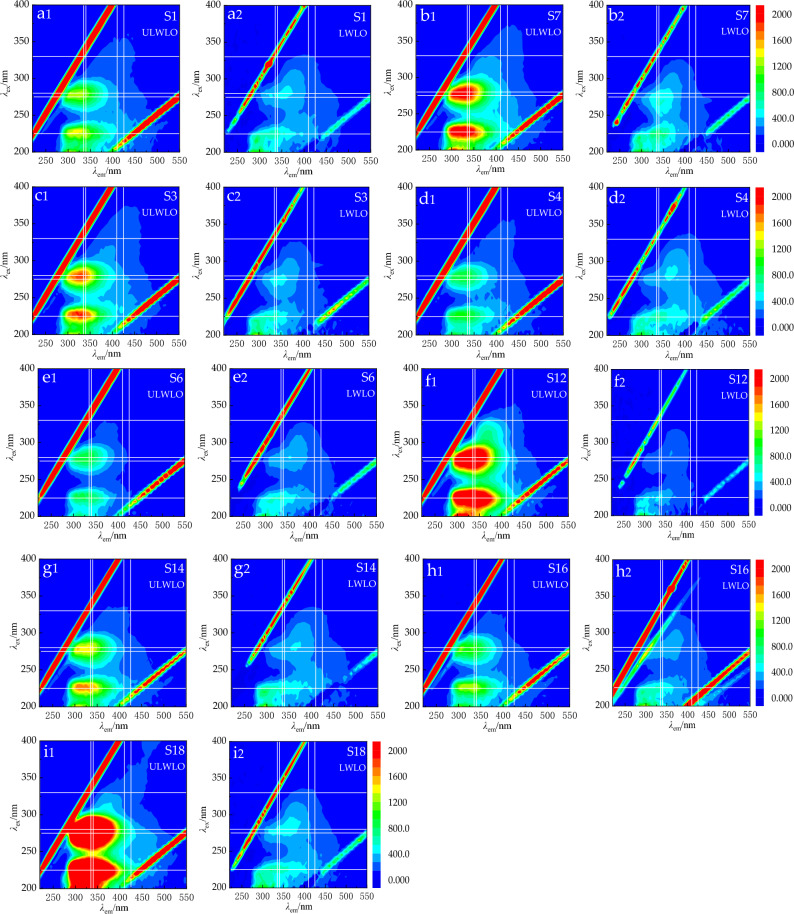
3D fluorescence spectra of DOM before and during low-water operation (**a1**–**b2**) Reservoir upstream; (**c1**–**d2**) Reservoir center; (**e1**–**f2**): Reservoir downstream; (**g1**–**h1**) Channel; (**i1**, **i2**) Point source pollution area).

As shown in Fig. 4a–i, five representative regions of the upstream of the reservoir (S1, S7), the core of the reservoir (S3, S4), the downstream of the reservoir (S12), the channel (S14, S16) and the point source contaminated area (S18) were selected for 3D-EEM analysis, and their fluorescence spectra plots were similar, all of them had obvious protein-like T1 peaks (= 225–230 nm/320–350 nm) and protein-like T2 peaks (= 270–285 nm/320–340 nm). The fluorescence response intensity of T1 and T2 peaks in the downstream of the reservoir and point source pollution area of Shahe Reservoir at the early stage of low water level operation was significantly higher than other areas. And a weak humic-like A peak (= 220–230 nm/380–440 nm) with C peak (= 280–310 nm/380–440 nm) formed by humic acid and fulvic acid together can also be observed in the downstream of the reservoir and point source pollution zone^36^. The fluorescence response intensities of T1 and T2 peaks in the point source pollution zone of Shahe Reservoir during low water level operation were significantly weakened and were not significantly different from those in the upstream of the reservoir, the core zone of the reservoir, and the downstream of the reservoir and the river. The fluorescence response intensities of T1 and T2 peaks in the downstream of the reservoir decreased the most and were smaller than those of the sampling points in other areas.

DOM was distinguished according to terrestrial and biogenic sources, terrestrial sources are formed by higher plant and animal residues in sediments degraded by fungi and bacteria, mostly showing absolute dominance of humic-like peaks; biogenic sources refer to biological activities generated by algae, plankton, and aquatic bacteria in the water column, mostly showing absolute dominance of protein-like peaks^37^.The dominance of protein-like T1 and T2 peaks of DOM in the three-dimensional fluorescence spectra indicates that the point source pollution area of Shahe reservoir and all other regions DOM originated from autogenous sources. Fluorescence index (FI), which is the ratio of fluorescence intensity at 370 nm excitation wavelength, 450 nm and 500 nm emission wavelength^38^, later corrected to 470 nm and 520 nm emission wavelengths^39^was used to characterize the source of humic substances in dissolved organic matter, greater than 1.9 indicates that it originated from processes such as microbial metabolism, less than 1.4 indicates a major contribution from terrestrial sources^40^. In this study, FI (370:470)/ FI (370:520) was used as the fluorescence index, and the fluorescence index in the interstitial water of sediment in all areas of the Shahe reservoir during the ULWLO ranged from 2.29 to 3.21, with a mean value of 2.72 ± 0.31, where the point source pollution zone (FI = 2.97), the river (FI = 3.17 ± 0.04), the upper reservoir (FI = 2.56 ± 0.01), reservoir core (FI = 2.38 ± 0.09), and reservoir downstream (FI = 2.64 ± 0.20); fluorescence index in sediment interstitial water in all areas of Shahe Reservoir during LWLO ranged from 1.98 to 2.35 with a mean value of 2.17 ± 0.14, of which, point source pollution area (FI = 2.30), river channel (FI = 2.15 ± 0.08), upstream of the reservoir (FI = 2.03 ± 0.02), the core area of the reservoir (FI = 2.30 ± 0.05), and downstream of the reservoir (FI = 2.15 ± 0.17), which indicates that DOM in all areas of the Shahe reservoir in both ULWLO and LWLO periods originated from authigenic sources of authigenic microorganisms, algae, etc.

**Figure 5:**
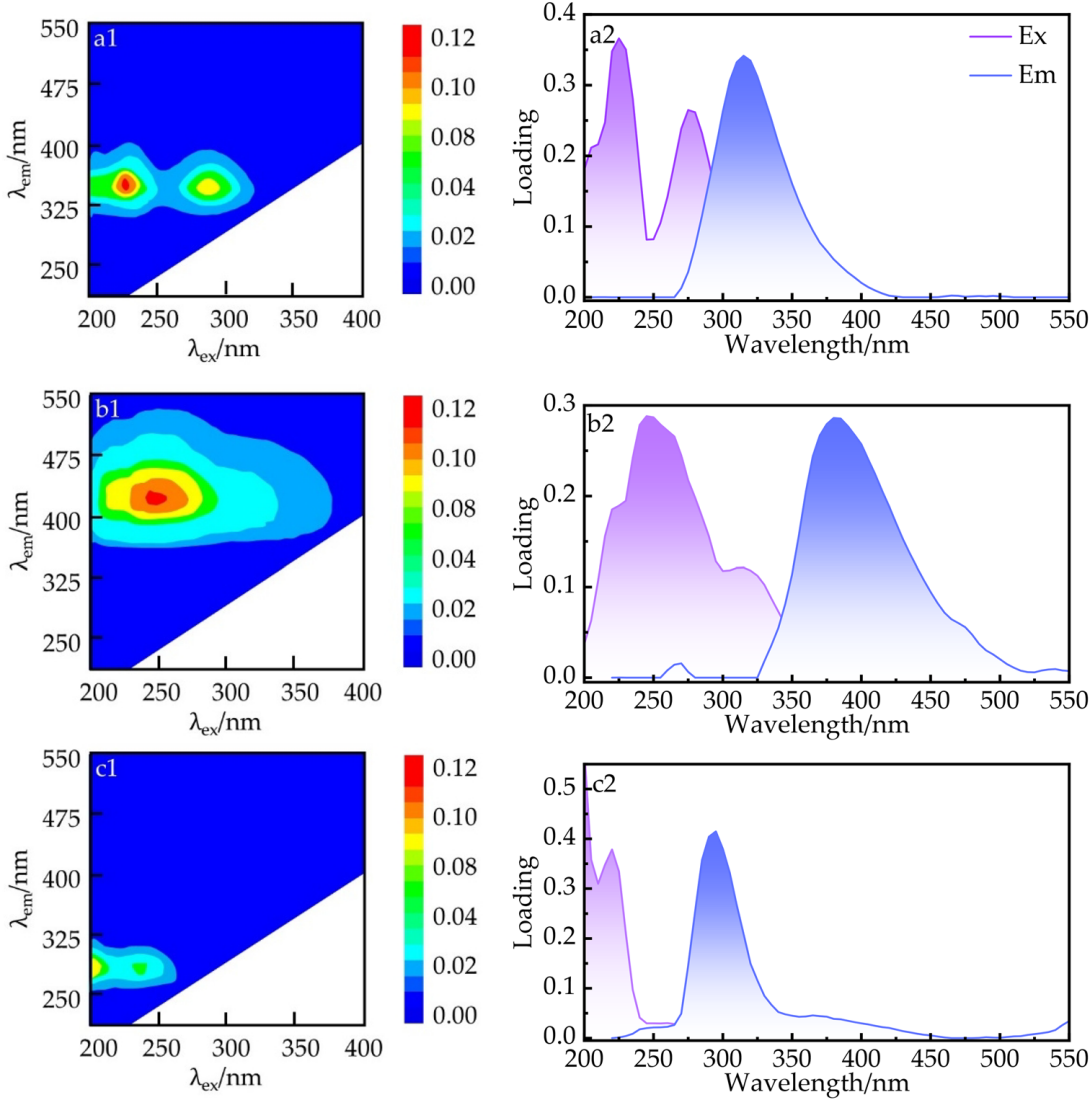
Fluorescent components by the PARAFAC and their excitation/emission wavelengths of DOM.

By decomposing the two sampled data through PARAFAC analysis, a total of three effective fluorescence components were identified, see Fig. 5, namely a1 (= 225, 275 nm/335 YIB), b1 (= 245 nm/410 nm), c1(= 220 nm/295 nm). The two peaks of the a1 component indicate low excitation light-like tryptophan (S) and high excitation light-like tryptophan (T), respectively; the b1 component indicates fulvic acid-like (A); and the c1 component indicates low excitation light-like tyrosine (D)^41^.

Fulvic acid (A) mainly originates from exogenous materials such as soil exudates, forest streams, leaf decomposing leaves, and biomass exudates; low excitation light-like tryptophan (S) and high excitation light-like tryptophan (T) are produced through microbial and bacterial degradation and metabolism, which can be free or bound proteins or amino acids^42^; Studies^43^ have shown that low excitation light-like tyrosine (D) is mainly derived from domestic wastewater and characterizes the less decomposed and fresher protein class. That is, low excitation phototyrosine (D), low excitation phototyrosine (S) and high excitation phototyrosine (T) are related to endogenous biological activities such as active bacteria, and can be used as indicators of changes in phytoplankton biological activities in the water column. It can be seen that components a1 and c1 mainly originate from endogenous pollution, and component b1 mainly originates from exogenous pollution.

The changes of DOM in the sediment of the Shahe Reservoir during the LWLO are shown in Table 3. The fluorescence peaks of tryptophan protein A (= 280 nm/335 nm), aromatic protein B (= 225 nm/340 nm), humic acid C (= 330 nm/410 nm) and fulvic acid D (= 275 nm/425 nm) in the sediment DOM were weakened. The fluorescence response intensity of tryptophan and aromatic proteins was weakened by 15.52 and 10.98 times, and that of humic acid and fulvic acid was weakened by 1.32 and 2.40 times, respectively. The next two sites were downstream (S12) and upstream (S7), where tryptophan, aromatic protein, humic acid and fulvic acid were weakened by 4.67, 1.59, 2.90, 1.87 and 4.57, 2.71, 2.64, 2.04 times, respectively. However, the fluorescence response of fulvic acid in the DOM of the core area (S4) and the downstream area (S6) increased by 1.13 and 1.45 times, and the fluorescence response of humic acid and fulvic acid in the DOM of the North Shahe River channel (S14) increased by 1.12 and 1.02 times, respectively. The attenuation intensity of fluorescence peaks A and B were higher than that of fluorescence peaks C and D during the LWLO of the Shahe Reservoir, indicating that high water flow rate and dissolved oxygen content attenuated the proteins in DOM more than humic acid and fulvic acid.

**Table 3:** Fluorescence intensity of EPS component during ULWLO and LWLO of Shahe Reservoir.

Partition	Sampling point	Normal water level operation period The fluorescence intensity				Low water level operation period The fluorescence intensity			
		Tryptophan protein	Amino acid protein	Humic acid	Furic acid	Tryptophan protein	Amino acid protein	Humic acid	Furic acid
Reservoir upstream	S1	1027.0	1282.0	185.1	236.8	415.8	638.9	141.4	206.4
	S7	2152.0	2289.0	248.3	315.9	471.2	844.1	94.2	155.0
Reservoir downstream	S3	1778.0	1725.0	232.8	310.1	389.0	582.1	91.9	135.0
	S4	930.8	881.1	172.1	204.1	332.3	763.2	167.9	230.5
Reservoir center	S6	947.4	828.0	113.1	118.8	361.2	590.6	99.8	172.1
	S12	1019.0	709.2	148.2	160.1	218.0	446.0	51.1	85.5
Channel	S14	1400.0	1513.0	141.8	213.7	381.1	195.2	158.6	217.9
	S16	1063.0	1328.0	112.0	174.5	320.1	519.0	87.7	168.0
Point source pollution area	S18	6897.0	6849.0	215.8	532.1	444.5	623.6	163.8	221.8

## Conclusions

1. The nutrient content in sediment and interstitial water of Shahe Reservoir increased significantly after LWLO: NH_4_^+^–N and TN in interstitial water increased by 0.78 and 2.34 mg·L^−1^, respectively; PO_4_^3−^–P and TP increased by 1.36 and 4.33 mg·L^−1^, respectively; TN and TP in sediment increased by 262.38 and 204.45 mg·kg^-1^, respectively.
2. Pearson correlation analysis and principal component analysis showed that OM was significantly correlated with nutrients: OM and TN (*p* < 0.01) and OM and TP (*p* < 0.05) in the pre-low water operation; OM and TN and TP (*p* < 0.01) in the low water operation.
3. The FI index indicates that the DOM in all areas of the Shahe reservoir before and after the low water operation was derived from authigenic sources of autotrophic microorganisms and algae.
4. PARAFAC analysis showed that the main organic pollutants in DOM were tryptophan-like (S, T), fulvic acid-like (A) and tyrosine-like (D). Among them, tryptophan (S, T) and tyrosine (D) mainly came from endogenous pollution,; fulvic acid (A) mainly came from exogenous pollution.

## Data Availability

The datasets used and analysed during the current study available from the corresponding author on reasonable request.
